# Treatment-seeking behaviour and associated costs for malaria in Papua, Indonesia

**DOI:** 10.1186/s12936-016-1588-8

**Published:** 2016-11-08

**Authors:** Muhammad Karyana, Angela Devine, Enny Kenangalem, Lenny Burdarm, Jeanne Rini Poespoprodjo, Ram Vemuri, Nicholas M. Anstey, Emiliana Tjitra, Ric N. Price, Shunmay Yeung

**Affiliations:** 1 National Institute of Health Research and Development, Ministry of Health, Jakarta, Indonesia; 2 Mahidol-Oxford Tropical Medicine Research Unit, Mahidol University, Bangkok, Thailand; 3 Nuffield Department of Clinical Medicine, Centre for Tropical Medicine and Global Health, University of Oxford, Oxford, UK; 4 Timika Malaria Research Program, Papuan Health and Community Development Foundation, Timika, Papua Indonesia; 5 Mimika District Health Authority, Timika, Papua Indonesia; 6 Department of Child Health, Faculty of Medicine, University Gadjah Mada, Yogyakarta, Indonesia; 7 Business School, Charles Darwin University, Darwin, Australia; 8 Global and Tropical Health Division, Menzies School of Health Research and Charles Darwin University, Darwin, Australia; 9 Department of Clinical Research, Faculty of Infectious and Tropical Disease, London School of Hygiene & Tropical Medicine, London, UK

**Keywords:** Malaria, Falciparum, Vivax, Malariae, Anaemia, Indonesia, Treatment-seeking, Cost, Primaquine, Adherence

## Abstract

**Background:**

Malaria remains a significant public health issue in Eastern Indonesia, where multidrug resistant *Plasmodium falciparum* and *Plasmodium vivax* are highly prevalent. The objective of this study was to describe treatment-seeking behaviour and household costs prior to a change to a unified treatment policy of dihydroartemisinin-piperaquine in Mimika district, Papua province in 2006.

**Methods:**

In 2005 a randomized cross-sectional household survey was conducted to collect data on demographics, socio-economic status (SES), treatment-seeking, case management, and household costs. Information on the cost of illness was also collected from patients exiting health facilities, in order to compare the cost of episodes diagnosed as *P. vivax* compared with those diagnosed as *P. falciparum*.

**Results:**

825 households were included in the survey. Of the 764 individuals who sought treatment for fever outside the home in the last month, 46% (349/764) went to a public health facility. Of the 894 reported visits to healthcare providers, 48% (433) resulted in a blood test, of which 78% (337) were reportedly positive. Only 10% (17/177) of individuals who reported testing positive for *P. falciparum* or mixed infection received the first-line treatment of chloroquine with SP, and 38% (61/159) of those with a diagnosis of *P. vivax* reportedly received the first-line treatment of chloroquine and primaquine. Overall, public facilities were more likely to prescribe the correct prevailing first-line drug combinations than private providers (OR = 3.77 [95% CI 2.31–6.14], p < 0.001). The mean cost to the household of an episode of *P. vivax* was similar to the cost of *P. falciparum* [US$44.50 (SD: 46.23) vs US$48.58 (SD: 64.65)].

**Conclusions:**

Private providers were a popular source of treatment for malaria, but adherence to the national guidelines was low and the economic burden of malaria for both *P. falciparum* and *P. vivax* infections was substantial. Engagement with the private sector is needed to ensure that patients have access to affordable good quality, effective diagnostics and anti-malarials for both *P. falciparum* and *P. vivax*.

**Electronic supplementary material:**

The online version of this article (doi:10.1186/s12936-016-1588-8) contains supplementary material, which is available to authorized users.

## Background

Great progress has been made in reducing the burden of malaria in the Asia Pacific region, however the ultimate goal of eliminating malaria has been undermined by the emergence and spread of drug resistance. The World Health Organization recommends that once anti-malarial efficacy falls below 90%, treatment policy should be changed with a combination therapy with greater than 95% efficacy [1].

The impact of any anti-malarial drug policy will depend on how it is implemented, and whether it ensures that individuals with malaria can easily access and receive the right treatment and diagnostics at an affordable cost. Early diagnosis of individuals with malaria and administration of effective treatment is critical to both to individual patients in ensuring rapid resolution of symptoms and preventing progression to severe disease, but also the community, by reducing further transmission and spread of anti-malarial drug resistance.

In many malaria-endemic countries, the private sector is an important source of care for individuals seeking treatment for malaria and most of the spending is out-of-pocket. The private sector comprises a diverse range of providers including private hospitals, registered pharmacies and unlicensed drug shops. Having an understanding of the role of different types of health care providers and engaging with them appropriately is, therefore, very important if changes in policy are to result in actual change in how patients with malaria are treated. Treatment-seeking behaviour of patients with symptomatic malaria is influenced by socio-economic factors, knowledge and beliefs, and access to healthcare provision. Malaria disproportionately affects rural poor households for whom one episode of illness can have catastrophic results, not only in terms of morbidity and mortality but also in terms of financial burden.

The literature on treatment-seeking behaviour for malaria, has shown that individuals often begin by taking treatment at home or from traditional healers and then move on to private drug sellers with few accessing public facilities directly [2, 3]. In sub-Saharan Africa, which carries the highest burden of malaria, studies have shown that less than 25% of the private providers have malaria tests available [4, 5]. In Asia, a recent household survey in Cambodia showed that two-thirds of individuals seek treatment at private providers [6]. Another survey in Myanmar revealed that nearly half of those who sought treatment did so at private providers where they were less likely to receive a diagnostic test for malaria and more likely to receive anti-malarials without a diagnosis [7].

The greatest burden of malaria in Indonesia is in the eastern provinces including Papua, where *Plasmodium falciparum* and *Plasmodium vivax* are the dominant species [8, 9]. Malaria outbreaks are frequent, often triggered by the migration of non-immune individuals from low to high transmission areas [10]. Prior to 2006, the first-line treatment of uncomplicated *P. falciparum* malaria was chloroquine (CQ) plus sulfadoxine-pyrimethamine (SP) and that for patients with uncomplicated *P. vivax* was CQ with 14 days of unsupervised primaquine (total dose 3.5 mg/kg over 14 days), for patients over 1 year of age [11]. A series of anti-malarial efficacy studies conducted in 2004 and 2005, documented high levels of resistance to both CQ and SP [12, 13].

At the time of this household survey, little data were available on treatment-seeking behaviour, anti-malarial drug use and household costs of treatment for malaria in Indonesia, where malaria remains a significant public health concern [14, 15]. A household survey in 2002–2003 indicated that less than 1% of children under the age of five received an anti-malarial for fever [15]. Another household survey conducted in Java in 2006 showed that the public sector was the primary source of malaria treatment for less than half of those with self-reported malaria [14]. Although several treatment-seeking surveys have been conducted in Asia [6, 7, 14, 16–22], almost no data exists on the associated healthcare costs for *P. falciparum* and *P. vivax* malaria in a co-endemic area as only one reported costs associated with each malarial episode with little detail on what these costs entailed [14].

The aim of the current study was to describe treatment-seeking behaviour for fever and associated costs of *P. vivax* and *P. falciparum* infection in Papua, prior to March 2006, when policy was changed to dihydroartemisinin-piperaquine (a fixed dose combination therapy) for uncomplicated malaria due to any species of malaria. A subsequent household survey to determine changes in treatment seeking and costs was conducted in 2013 and will be reported separately.

## Methods

### Site details

The survey was conducted in Mimika Regency, southern Papua, Indonesia. This district covers an area of 21,522 km^2^ with approximately 130,000 people living in 12 districts in 2004. Most of the population resides in Timika town and the surrounding villages. The area is inhabited by a variety of ethnic groups, which are broadly categorized as lowland Papuans, highland Papuans or non-Papuans. The demographics, malaria transmission and health services in this region have been described previously [8].

The area is covered with thick rain forest with both coastal and mountainous areas. Malaria transmission occurs throughout the year and is restricted to the lowland area where most of the population lives. Immigration to work at the local mine is considerable and has the potential both to expose non-immune individuals to malaria and to introduce malaria from other areas of Indonesia. There are three mosquito vectors: *Anopheles koliensis*, *Anopheles farauti* and *Anopheles punctulatus.* Based on the malaria surveillance data, the annual incidence of clinical malaria, is estimated to be approximately 876 episodes per 1000 people, with a slight predominance of *P. falciparum* infection (46%) compared to *P. vivax* (39%) [8].

Healthcare provision is provided free of charge at the hospital, 12 Government-funded Primary Health Clinics (Puskesmas) and, for those employed by a local mining company, at the malaria control clinics where parasitological diagnosis by microscopy and rapid diagnostic test are available. There is also a thriving private sector, which includes clinics, pharmacies and drug stores. Whilst anti-malarials are officially prescription drugs, they can also be purchased over the counter at the private sector [10]. The malaria surveillance programme does not capture the malaria cases seen in the private sector.

### Household sampling strategy

The survey was conducted in the Mimika district between July and December 2005.

The sample size calculation aimed to estimate the true prevalence of households with someone with a recent history of fever and was based on an estimated prevalence of 40% and population size of 140,000; a multiplication factor of 2 was used to take into account the design effect due to cluster sampling. A sample size of 800 households was required to achieve an estimate of prevalence with greater than 95% confidence.

A three-stage cluster sampling procedure was applied to identify 32 clusters of 25 households [23]. Firstly the four most populous sub-districts (Mimika Baru, Mimika Timur, Kuala Kencana and Tembaga Pura) were chosen from the 12 sub-districts based on their accessibility by road from the main town of Timika and because they included the majority (80%) of the district’s population. Secondly, the number of clusters in each sub-district was apportioned according to their relative populations. The houses were then assigned numbers according to their geographical location and clusters of 25 houses were identified randomly according to WHO guidelines with the first house identified by random allocation.

Members of the households were defined as all individuals living under one roof, who ate from one kitchen and had resided in the study area for at least six months. If a selected household was empty, the interviewer returned the next day. If still unoccupied, the next occupied household was sampled instead. Temporary migrants were defined as individuals who had moved into the area within last six months and did not plan to stay longer than six months; these were excluded from the survey.

### Data collection

The survey was conducted using a structured questionnaire capturing self-reported household demographic data, income, average monthly food and non-food expenditures, expenditure on healthcare in the last month, ownership of assets, and bed net ownership and use. Nearly all surveys were conducted in the local language, Bahasa Indonesian. The survey was conducted with the head of the household or another suitable adult and details on all household members gathered. Those members who were present were asked specific details about recent illness, had a physical examination and were asked to consent to a blood film as previously described [8].

In order to obtain detailed information about the care-seeking process and cost of illness, individuals reporting a history of fever in the preceding 30 days were asked to complete a separate module on treatment-seeking, comprising of questions on all the places that they went to seek treatment, whether they received a blood tests and the type of medicines they received. They were also asked to about direct and indirect household costs including the cost of treatment, transport, productivity losses due to illness, and companion, caretaking and substitute labour [21].

An exit survey was also undertaken on patients with confirmed malaria as they left public facilities in order to collect accurate information on household costs in relation to type (species) of malaria infection. The exit interviews were carried out over a period of four months in the hospital, two of the four Puskesmas and three of the eight malaria control clinics. The number of interviews per type of facility was proportional to the number of malaria patients each facility saw each month. Any patient with a parasitological diagnosis of malaria was eligible for inclusion. Patients completed a questionnaire with the aid of a research nurse to document treatment-seeking and costs of illness. With a shorter time between the event and the survey, these results were less likely to be confounded by recall bias and enabled a greater certainty about malaria species diagnosis with the disadvantage of truncating the costs at the end of the healthcare visit.

### Data analysis

Data were analysed using STATA statistical software (version 14) [24] and R (version 3.2.3) [25]. Frequencies and percentages were used for the descriptive data. Percentages were compared using the Chi square test (X^2^). Differences in outcome distributions were tested using Mann–Whitney test for two groups and the Kruskal–Wallis H test for more than two groups. Continuous variables were compared using Spearman’s rank for correlation. Simple logistic regression was used to calculate odds ratios (ORs). The variables that were significant risk factors (p < 0.05) for fever in the past month in the univariate analysis were included in a multiple logistic regression model. Regression models involving behaviour of the individuals were adjusted for clustering by households, since more than one household member could have been present for each household.

For the analysis on treatment-seeking behaviour, those who reported that their fever started in the last 2 days were excluded in order to ensure that only individuals who had had sufficient time to seek or not seek treatment were included. For those included in the treatment-seeking behaviour analysis the following outcomes were calculated: the percentage that reported taking any medicines (including at home or traditional medicine) and percentage seeking treatment outside of the home according to the number and type of provider, categorised into private providers (private clinics, pharmacies and drug shops) and public facilities (the hospital, Puskesmas and malaria control clinics).

In order to explore how patients were managed at each provider in terms of blood testing and prescriptions of anti-malarials, further analysis was undertaken on each patient-provider interaction (“visit”). As anti-malarial injections pose the risk of infection to patients and are largely inappropriate treatments for uncomplicated malaria, individuals were also asked whether they received an injection or tablets or both.

In order to categorise the surveyed by socio-economic status, a Discriminant Analysis of Principal Components [26] was used to analyse the data on reported household ownership of assets. This approach maximized between group variability while minimizing within group variability, capturing heterogeneity in ownership that was not seen using a standard principal component analysis [27]. In order to investigate the relationship between SES and other outcomes captured by the survey, the groups were ranked from poorest to richest in terms of both income and expenditure per household member.

Costs were gathered in Indonesian rupiah (IDR), and converted into United States Dollars (US$) using the average exchange rate for 2005 [28] and then revised to the 2014 equivalent using the consumer price index for Indonesia [29]. To represent the cost burden on households, the mean and standard deviation (SD) are reported. As the data were skewed, the Mann–Whitney test was used for comparisons; the median and interquartile range (IQR) are included in the Additional files. Direct costs, including consultation, diagnosis, medication, overnight stays, administration and transportation, were reported according to where treatment was obtained [30]. Indirect costs, including lost wages for companions, caretakers and substitute labourers, are aggregated into cost per individual taking treatment. As a result, when multiple companions, caretakers or substitute labourers were reported for a single individual taking treatment, their times were summed, resulting in the mean days per individual taking treatment. The mean wage was calculated for all who reported a wage, including individuals taking treatment, companions, caretakers and substitute labourers. This wage was applied to all who reported reducing their activities because they had a fever or were a companion, caretaker or substitute labourer for someone with a fever. For those who reported reducing some of their usual activities, the days of lost activity were multiplied by one half day’s wage and included in the total costs.

## Results

### Household demographics, SES and expenditure

**Fig. 1:**
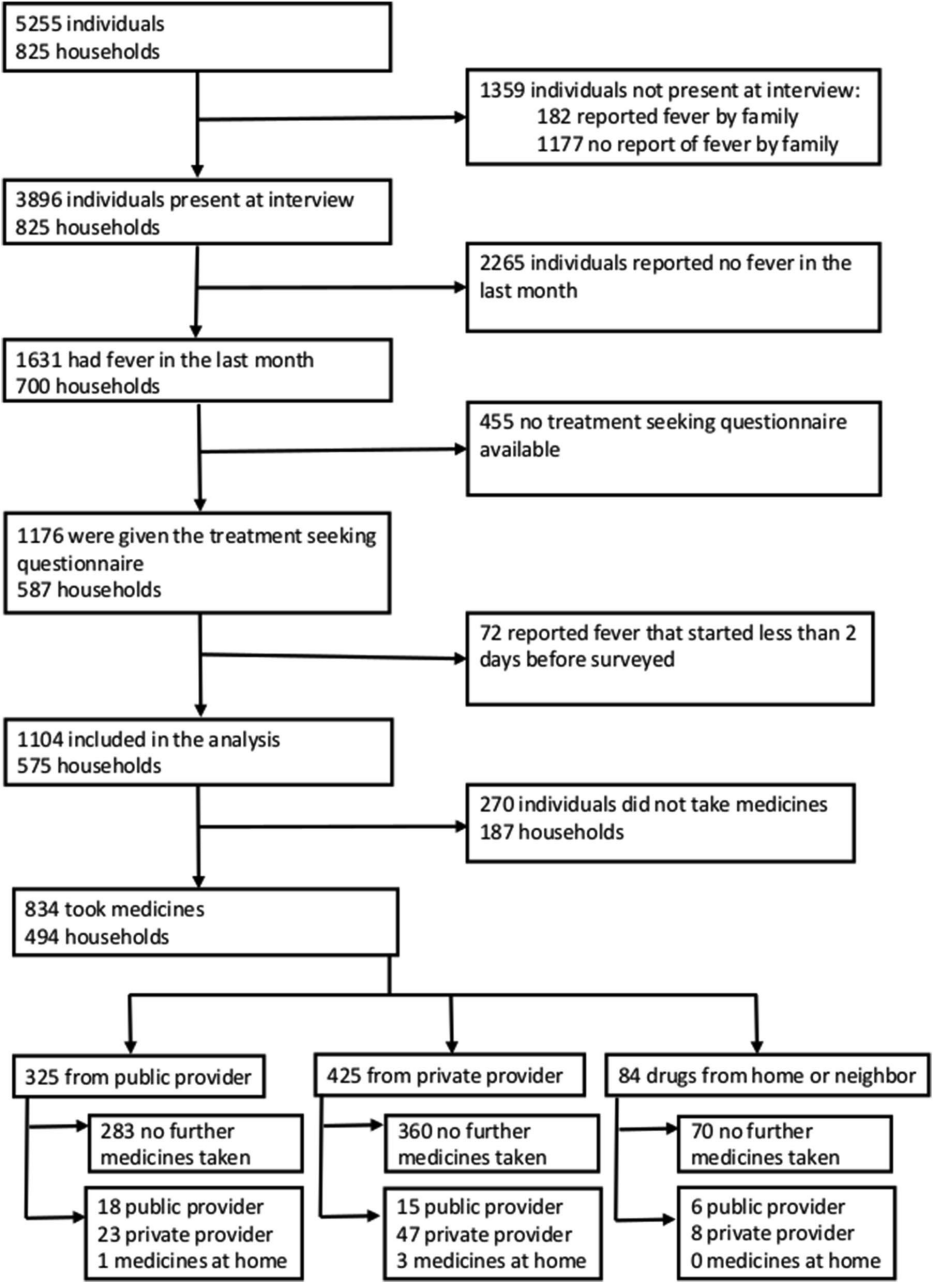
Flow diagram. Diagrammatic presentation of household members, interviews, initial location of treatment taking, and whether took a second treatment

In total 825 households with 5255 individuals were included in the survey between July and December 2005 (Fig. 1). The median household size was six individuals (IQR 4–8) with a maximum of 24 in one house. The median duration that a household had lived at that location was nine years (IQR 4–15). Thirty-four (4%) of households reported moving into that location less than a year previously. Of the 763 households in which income was recorded, 63% (482) reported a total monthly income of less than US$500.

For the SES, the Discriminant Analysis of Principal Components resulted in households being split into five groups ranging in size from 111 to 203. Richer households had electricity and owned electrical items but did not always own a house or land, whereas poorer households tended to own houses, land and grow crops, but were less likely to have electricity (Additional file 1: Figure S1). Household SES was significantly lower in Highland Papuan household with 47% (101/216) of Highland Papuans in the poorest categories compared to 12% (22/190) of Lowland Papuans and 4% (16/416) of non-Papuans (p < 0.001). The household general health expenditure in the previous month was significantly lower in households with lower SES (p = 0.001; Additional file 2: Figure S2). The estimated household general health expenditure was correlated with the number of individuals reporting fever in a household (ρ = 0.209, p < 0.001).

Only 340 (41%) of households reported owning a bed net, and this was inversely correlated with SES with 51% (72/140) in the poorest group reported bed net ownership as compared to 29% (32/111) in the richest (p < 0.001).

### History of fevers and parasite positivity of household members

In total 3896 (74%) of individuals were present at the time of interview (Fig. 1) of whom 48% (1877) were male, 42% (1644) were under the age of 15 (Table 1), and 53% (2069) were Papuan. In total, 1631 (42%) of individuals from 700 (85%) households reported having had a febrile illness in the preceding month. Significant independent risk factors for febrile illness included age, ethnicity, and poor economic status (Table 2).

**Table 1:** Demographic characteristics of individuals in surveyed households

Characteristic	All household members (N = 5255)	Present during survey (N = 3896)	Reported fever during previous month (N = 1631, 42%)	Reported fever starting >2 days before survey and took treatment (N = 834, 51%)
*Age (years)*—*n (%)*				
0–4	870 (17%)	820 (21%)	405 (25%)	205 (25%)
5–14	1101 (21%)	824 (21%)	306 (19%)	148 (18%)
15+	3284 (62%)	2252 (58%)	920 (56%)	481 (58%)
Sex (female)—n (%)	2409 (46%)	2019 (52%)	850 (52%)	433 (52%)
Pregnant (yes)—n (%)	92 (2%)	87 (2%)	39 (2%)	23 (3%)
*Place of birth*—*n (%)*				
Highland Papuan	1494 (28%)	1045 (27%)	385 (24%)	172 (21%)
Lowland Papuan	1371 (26%)	1024 (26%)	463 (28%)	183 (22%)
Non-Papuan	2390 (45%)	1827 (47%)	783 (48%)	479 (57%)
Resided in lowlands for more than 1 year? (yes)—n (%)	4969 (95%)	3674 (94%)	1547 (95%)	797 (96%)
*District*—*n (%)*				
Mimika Baru	4401 (84%)	3242 (83%)	1401 (86%)	740 (89%)
Mimika Timur	371 (7%)	286 (7%)	108 (7%)	11 (1%)
Kuala Kencana	331 (6%)	249 (6%)	93 (6%)	65 (8%)
Tembaga Pura	152 (3%)	119 (3%)	29 (2%)	18 (2%)

**Table 2:** Logistic regression analysis of risk factors for reporting a fever in the past month (N = 3896)

Variable	OR (95% CI)	p value	AOR (95% CI)	p value
*Gender*				
Male	Reference		Reference	
Female	1.02 (0.90–1.16)	0.756	–	–
*Age (years)*				
<5	1.47 (1.26–1.73)	<0.001	1.49 (1.27–1.74)	<0.001
≥5	Reference		Reference	
*Pregnant*				
No	Reference		Reference	
Yes	1.13 (0.74–1.73)	0.566	–	–
*Household size*				
≤7 members	1.16 (0.98–1.38)	0.091	–	–
>7 members	Reference		Reference	
*SES groups*				
Richest	Reference		Reference	
Fourth	1.47 (1.11–1.95)	0.007	1.47 (1.11–1.96)	0.007
Middle	1.33 (1.00–1.76)	0.050	1.40 (1.05–1.87)	0.022
Second	1.17 (0.89–1.53)	0.270	1.35 (1.01–1.80)	0.046
Poorest	1.52 (1.15–2.00)	0.003	2.04 (1.48–2.81)	<0.001
*Ethnicity*				
Highland Papuan	Reference		Reference	
Lowland Papuan	1.41 (1.12–1.78)	0.003	1.64 (1.28–2.10)	<0.001
Non-Papuan	1.29 (1.06–1.55)	0.009	1.59 (1.26–2.01)	<0.001
*Resided in lowlands* >*1* *year?*				
No	Reference		Reference	
Yes	1.19 (0.86–1.66)	0.292	–	
*Family sleeps under a bed net?*				
No	Reference		Reference	
Yes	1.02 (0.86–1.21)	0.792	–	

The multiple logistic regression included variables that were significant (p < 0.05) in the univariate logistic regression

In those who consented to blood testing, malaria slide positivity was 16% (634/3890) of whom 46% (290) were infected with *P. falciparum*, 39% (248) with *P. vivax*, 11% (72) with mixed infections and 4% (24) with *P. malariae.* Individuals in the lower three SES groups were more likely to be parasite positive than those in the top two SES groups (OR = 1.33 [95% CI 1.05–1.68], p = 0.019).

### Treatment-seeking behaviour of household members

Treatment-seeking behaviour was assessed in 72% (1176/1631) of individuals who reported fever in the preceding month (Fig. 1). The analysis of treatment-seeking was restricted to the 94% (1104/1176) of individuals with a fever commencing at least two days before the survey (Fig. 1). Three-quarters (76%, 834/1104) reportedly took some form of medicine. For the 270 (24%) of participants who did not take any medication, the most common explanation offered was that the disease was not severe enough to warrant it (169, 63%).

Of those who took some form of medicine, 92% (764/834) sought treatment outside of the home at some point. Treatment-seeking outside of the home was significantly more likely in those who had a febrile illness lasting two or more days (OR = 2.97 [95% CI 2.15–4.09], p < 0.001), were in the richest two SES groups (OR = 1.99 [95% CI 1.42–2.78], p < 0.001) and were non-Papuan (OR = 2.63 [95% CI 1.92–3.60], p < 0.001). Treatment-seeking was not influenced by age or gender. Of those who sought treatment outside of the home, 46% (349/764) went to a public provider at least once; this percentage being greater in those who were female, less than 15 years old, Papuan ethnicity or from the poorest SES (Table 3).

**Table 3:** Sociodemographic characteristics associated with whether ever sought treatment at a public provider (N = 764)

Variable	Source of treatment [% (n)]		Univariate OR (95% CI)	p value
	Private provider(s) only (N = 415)	Public provider ever (N = 349)		
*Gender*				
Male	60% (219)	40% (148)	Reference	
Female	49% (196)	51% (201)	1.52 (1.14–2.02)	p = 0.004
*Age group (years)*				
<5	49% (93)	51% (98)	1.53 (1.06–2.21)	p = 0.024
5–15	46% (61)	54% (71)	1.69 (1.13–2.53)	p = 0.011
15+	59% (261)	41% (180)	Reference	
*Ethnicity*				
Non-Papuan	67% (288)	33% (142)	Reference	
Papuan	38% (127)	62% (207)	3.31 (2.31–4.73)	p < 0.001
*SES quintile*				
Poorest	31% (36)	69% (81)	7.03 (3.82–12.95)	p < 0.001
Second	46% (80)	54% (95)	3.71 (2.03–6.78)	p < 0.001
Middle	58% (100)	42% (73)	2.28 (1.25–4.16)	p = 0.007
Fourth	59% (99)	41% (68)	2.14 (1.17–3.93)	p = 0.013
Highest	76% (100)	24% (32)	Reference	

This is among fever patients who sought treatment outside of the home

OR = Odds Ratio; 95% CI = 95% confidence intervals

The 764 individuals who sought treatment outside of the home reported a total of 894 visits to healthcare providers. Further analyses were undertaken on these 894 interactions with providers.

### Diagnosis of malaria at healthcare providers

Out of the 894 visits to health care providers, a blood test for malaria was reportedly undertaken in 433 (48%) of these visits (Table 4) of which 78% (337) were reportedly positive: half (167) *P. falciparum*, 47% (159) *P. vivax*, 3% (10) mixed infections and 0.3% (1) *P. malariae*. For 3% (12/433) of visits, no result was reported. Children who were 14 years old or younger were more likely to have a blood test compared to individuals over the age of 14 (OR 1.42 [95% confidence interval (CI) 1.10–1.84], p = 0.007), although they were less likely to have a detectable parasitaemia (OR 0.30 [95% CI 0.18–0.51], p < 0.001).

**Table 4:** Percentage of respondents who reported receiving blood tests, anti-malarials and associated costs (N = 894)

Healthcare provider	Percentage with blood test for malaria n (%)	Frequency anti-malarials were prescribed n (%)	Visit costs	Transport costs	Total direct costs
			Mean (SD)	Mean (SD)	Mean (SD)
*Private (N* = *519)*	148 (29%)	300 (58%)	11.52 (29.86)	0.93 (1.96)	12.46 (30.25)
Private clinic or doctor (N = 240)	124 (52%)	187 (78%)	21.66 (41.44)	1.53 (2.73)	23.19 (41.77)
Pharmacy (N = 173)	23 (13%)	87 (50%)	3.76 (5.26)	0.58 (0.39)	4.34 (5.29)
Shop (N = 106)	1 (1%)	26 (25%)	1.25 (1.44)	0.15 (0.32)	1.40 (1.57)
*Public (N = 375)*	285 (76%)	247 (66%)	2.63 (13.87)	0.88 (1.66)	3.52 (14.33)
Puskesmas (N = 155)^†^	102 (66%)	94 (61%)	3.40 (11.20)	0.57 (0.69)	3.97 (11.57)
Malaria control clinic (N = 149)^††^	123 (83%)	107 (72%)	0.00 (0.00)	0.57 (1.59)	0.57 (1.59)
Hospital (N = 71)	60 (85%)	46 (65%)	6.49 (26.86)	2.24 (2.46)	8.72 (27.41)
p value (private vs public)	*<0.001* ^a^	*0.015* ^b^	*<0.001* ^c^	0.080^c^	*<0.001* ^c^

Mean cost and standard deviation (SD) by type of healthcare provider are reported in US$. Visit costs includes costs of consultation, diagnosis, medications and any other costs directly related to the care received. Transport cost includes costs for the patient and anyone who accompanied him or her

^a^OR = 7.94 [95% CI 5.86–10.76]

^b^OR = 1.41 [95% CI 1.07–1.86]

^c^Mann–Whitney test

^†^Government-funded primary health clinic

^††^Mining company-funded clinic

### Anti-malarial treatment at healthcare providers

An anti-malarial (alone or with other medicines) was reportedly prescribed during 61% (547/894) of visits: 86% (471/547) of which were prescribed as tablets, 5% (30/547) as an injection and 8% (46/547) as both an injection and tablets. Overall, public facilities were significantly more likely to prescribe anti-malarial treatment than private providers (OR = 1.41 [95% CI 1.07–1.86], p = 0.015); however, the majority (92%, 70/76) of anti-malarial injections were administered by private clinics or doctors.

In relation to parasitological testing prior to anti-malarial prescription, 61% (336/547) of the anti-malarial prescriptions followed a blood test. The corresponding figures were 87% (215/247) at public facilities and 40% (121/300) at private providers (p < 0.001).

Only 10% (17/177) of individuals who reported testing positive for *P. falciparum* or mixed infection reported receiving the prevailing first-line treatment of chloroquine with SP, with the majority receiving primaquine (129; 73%), chloroquine (103; 58%) and/or quinine (49; 28%). Those reporting a diagnosis of *P. vivax* were more likely to receive the correct first line treatment of chloroquine and primaquine [38% (61/159); Table 5]. Overall, public facilities were more likely to prescribe the correct drug combinations (OR = 3.77 [95% CI 2.31–6.14], p < 0.001).

**Table 5:** Anti-malarial treatment received according to blood test results as reported in the household survey

	Private clinic or doctor	Pharmacy	Shop	All private	Puskesmas^†^	Malaria control clinic^††^	Hospital	All public	Overall
Not tested (N)	116	150	105	371	53	26	11	90	461
% (n) anti-malarials^a^	69% (80)	49% (73)	25% (26)	48% (179)	43% (23)	35% (9)	0% (0)	36% (32)	46% (211)
Tested negative (N)	16	5	1	22	28	21	13	62	84
% (n) anti-malarials^a^	19% (3)	0% (0)	0% (0)	14% (3)	0% (0)	5% (1)	0% (0)	2% (1)	5% (4)
*P. falciparum* (N)	57	11	0	68	29	50	20	99	167
% (n) anti-malarials^ab^	98% (56)	73% (8)	–	94% (64)	97% (28)	100% (50)	95% (19)	98% (97)	96% (161)
% (n) CQ*^b^	51% (29)	27% (3)	–	47% (32)	79% (23)	88% (44)	5% (1)	69% (68)	60% (100)
% (n) SP*^b^	5 (9%)	0% (0)	–	7% (5)	0% (0)	28% (14)	0% (0)	14% (14)	11% (19)
% (n) PQ^b^	68% (39)	27% (3)	–	62% (42)	83% (24)	92% (46)	55% (11)	82% (81)	74% (123)
% (n) quinine^b^	33% (19)	36% (4)	–	34% (23)	17% (5)	6% (3)	75% (15)	23% (23)	28% (46)
% (n) anti-malarial injection^b^	32% (18)	9% (1)	–	28% (19)	3% (1)	0% (0)	10% (2)	3% (3)	13% (22)
*P. vivax* (N)	44	7	0	51	41	43	24	108	159
% (n) anti-malarials^ab^	95% (42)	86% (6)	–	94% (48)	100% (41)	98% (42)	100% (24)	99% (107)	97% (155)
% (n) CQ*^b^	34% (15)	71% (5)	–	39% (20)	68% (28)	77% (33)	8% (2)	58% (63)	52% (83)
% (n) PQ*^b^	34% (15)	0% (0)	–	29% (15)	78% (32)	81% (35)	88% (21)	81% (88)	65% (103)
% (n) quinine^b^	34% (15)	14% (1)	–	31% (16)	24% (10)	5% (2)	88% (21)	31% (33)	31% (49)
% (n) anti-malarial injection^b^	11% (5)	0% (0)	–	10% (5)	0% (0)	0% (0)	4% (1)	1% (1)	4% (6)

^a^Includes tablets and injections

^b^Medication categories are not mutually exclusive

^†^Government-funded primary health clinic

^††^Mining company-funded clinic

* Recommended treatment regimen

**Fig. 2:**
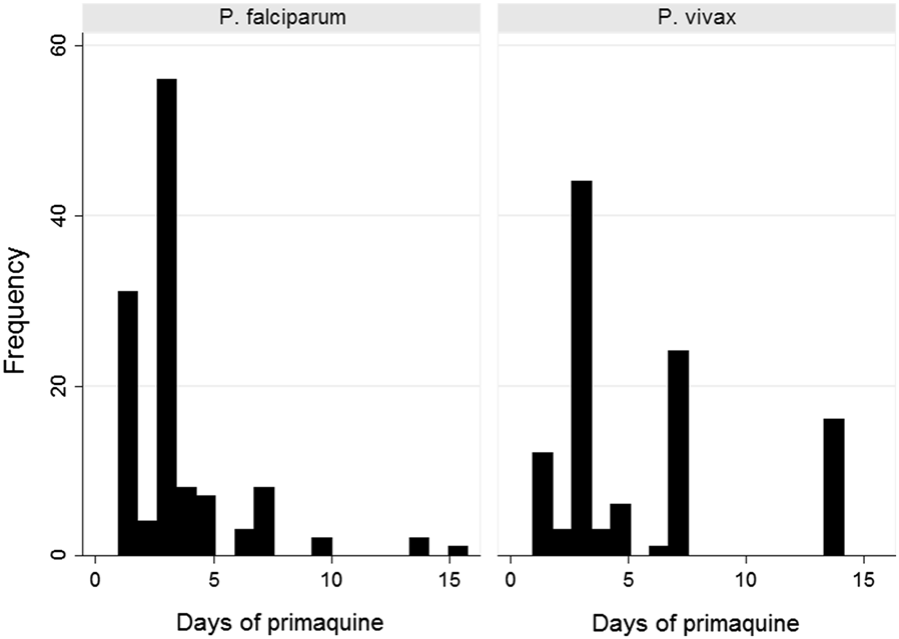
Histogram of primaquine treatment. Reported number of days of primaquine treatment for *P. falciparum* and *P. vivax*/mixed infections (n,%)

In total, 69% (232/337) of respondents who reported a positive blood test for malaria, reported being prescribed primaquine. Of those who reported taking primaquine for *P. vivax* infection (alone or mixed), 63% (69/109) reported taking less than 7 days of treatment (Fig. 2).

### Direct costs of treatment-seeking

The mean total direct cost to the household per visit at a private provider was US$12.46 (SD = 30.25) compared to US$3.52 (SD = 14.33) at the public sector (p < 0.001). Table 4 shows the mean cost of treatment (including drugs, consultation and diagnosis) and transport (including patient and any accompanying people) by source of treatment; Additional file 3: Table S1 shows the corresponding median treatment costs. No significant difference in direct costs were seen for those diagnosed with *P. falciparum* as compared with *P. vivax* infection. The total direct costs per person according to age group are presented in Additional file 4: Table S2.

### Indirect costs per fever episode

Overall, 77% (641/834) of those who reported taking treatment stated that at least one person had reduced his or her usual activities to care for them and 66% (553/834) of respondents reported having at least one companion with them when seeking treatment. Eight percent (66/834) of respondents reported the need for at least one substitute labourer to perform their usual activities while sick and this was significantly more likely in non-Papuans compared to Papuans (OR = 2.09 [95% CI 1.19–3.65], p = 0.010). Of those who reported a daily wage, the mean was US$10.92 (SD = 8.22). The days lost by the individual and his/her companions, caretakers and substitute labourers are shown in Additional File 4: Table S2 along with their associated costs. The total mean indirect cost per fever episode requiring treatment taking was US$31.49 (SD = 35.92) for children and US$53.52 (SD = 58.06) for adults.

### Malaria costs from healthcare facility exit survey

A total of 358 patients were surveyed after exiting six public health care facilities (the hospital, 2 Puskesmas and 3 malaria control clinics). The reported diagnosis was *P. falciparum* in 55% (196), *P. vivax* in 38% (136), mixed infection in 4% (12), *P. malariae* in 4% (13) and *P. ovale* in 0.3% (1). The direct and indirect costs related to visits at public providers for *P. falciparum* and *P. vivax* are presented in Table 6 and Additional file 5: Table S3. The total costs were similar between the species of diagnosis, however respondents with *P. falciparum* infection reportedly spent more money than those with *P. vivax* for the healthcare visit (p = 0.013), personal transportation to the healthcare facility (p < 0.001) and lost wages for the patient (p = 0.001). Individuals with *P. vivax* infection spent more money on companion costs (p = 0.034). When the analysis was restricted to children or adults, the only cost that remained statistically significant was for the healthcare visit.

**Table 6:** Patient costs per visit in US$ from facility exit surveys, mean (standard deviation)

	*P. falciparum* (n = 196)	*P. vivax* (n = 136)	p value
*Total direct costs*	2.69 (4.67)	2.09 (3.35)	0.056
Visit cost	0.90 (3.08)	0.40 (0.53)	*0.013*
Transport cost for patient	0.61 (1.41)	0.40 (1.24)	<*0.001*
Transport cost for companions	1.18 (2.53)	1.28 (2.61)	0.541
*Total indirect costs*	45.90 (63.10)	42.41 (44.98)	0.960
Lost wages for patient^a^	17.23 (29.73)	8.79 (19.59)	*0.001*
Lost wages for companions	5.46 (7.28)	6.95 (7.33)	*0.034*
Lost wages for caretakers	22.04 (40.55)	26.51 (36.39)	0.089
Lost wages for substitute labourers	1.25 (6.85)	0.16 (1.87)	0.096
Total costs	48.58 (64.65)	44.50 (46.23)	0.981

Visits were to public providers only and reported by malaria diagnosis

The per day wage was taken from the household survey (US$10.93). Mixed infections are excluded

Italic values indicate significance of p value (p < 0.05)

^a^For children, lost wages are recorded as no cost

## Discussion

This study from Papua, Indonesia, was conducted in an area with one of the greatest levels of malaria burden in the region and at a time of high levels of anti-malarial drug resistance to prevailing first-line therapies. It is the first study from eastern Indonesia reporting household treatment-seeking behaviour and is one of the few studies comparing household costs of *P. vivax* malaria with *P. falciparum* malaria, as well as self-reported adherence to 14 day primaquine.

There were several significant findings of relevance to malaria control efforts. Firstly in relation to treatment seeking, almost a third of individuals reporting a fever in the last month did not seek treatment outside of the home. This figure is higher than previous reports from central Java where 88% of households reported seeking treatment or advice outside of the home for their most recent malaria illness [14]. Over half (54%) of individuals sought treatment only in the private sector, despite the availability of free treatment in the public sector. Those who sought care in the private sector were less likely to report a diagnosis of malaria, but almost 60% still reported receiving anti-malarial treatment. Fifteen percent of individuals with fever reported more than one source of treatment, a likely consequence of the initial treatment failing to improve their symptoms. These findings highlight the importance of engaging with private sector in malaria control efforts and of the need to understand and address the reasons underling patient’s preferences.

Secondly in relation to anti-malarial treatment, of those patients who reported positive blood tests for malaria, few were prescribed the recommended drug regimen for the species that they reported; and for patients reporting *P. vivax* infections who reportedly received primaquine, over half of participants reported taking less than three days of the recommended 14 day regimen, a duration ineffective in preventing relapse [31].

Furthermore, primaquine was not recommended for *P. falciparum*, but the majority of patients reported receiving it. With such a large proportion of *P. falciparum* patients reporting primaquine use, it seems unlikely that this is entirely due to patient recall bias in terms of the species diagnosed or the drug prescribed. Such discrepancies in anti-malarial policy and actual practice have been described previously in other settings [32, 33]. There are many possible reasons including lack of provider awareness or understanding, patient and provider preferences, perceptions and experiences with regards efficacy and side effects. Understanding and addressing the reasons for these discrepancies is clearly important, especially in the face of increasing anti-malarial drug resistance.

Thirdly in relation to household costs, the household financial burden of fevers in this region was substantial and did not differ substantially by the species of malaria. The mean cost per fever episode reported in the household survey was (US$53.54) and included the direct costs of the whole illness episode, including subsequent visits to treatment providers, as well as any further indirect costs of time incurred after visits to a healthcare facility. The mean cost of visiting a public provider for *P. vivax* malaria was US$44.50, compared to US$48.58 for *P. falciparum*. This likely represents an underestimate of household costs as patients were at public facilities and not at the end of their malaria episode.

Over 60% of households reported a monthly income less than $500, indicating that a fever episode represented at least 11% of the household’s monthly income. This also represents nearly 5 h of work using the mean reported hourly wage of US$10.92. Individuals in poorer households were at greater risk of fevers, with additional indirect costs likely to accumulate in those who do not take effective treatment. These findings emphasize the enormous economic burden of recurrent *P. vivax* episodes on the most vulnerable communities.

Another strength of this study, is the use of Discriminant Analysis of Principal Components to create meaningful SES categories. In this setting, use of the traditional asset index would have resulted in misclassification of households into categories which would have not reflected a useful categorization of household’s actual status. Re-settled Papuans had been provided with house and land by the local government resulting in many of the poorer households having high levels of house and land ownership but little other disposable income. Lower SES status was a risk factor for both recent febrile illness and parasite positivity at the time of the survey. Those with lower SES status were more likely to ever seek treatment at a public facility, a likely reflection of the lower costs incurred. This, along with the higher likelihood of reporting fever in the past month by those with lower SES, highlights the heterogeneity of the population in relation to risk of fever and treatment-seeking behaviour.

This study has a number of limitations. Firstly, many of the older male household members were not present at the time of the survey and therefore not included in the analysis. In this region it is often the adult males who are at higher risk of malaria and so it is possible that we underestimated the true burden of fever episodes. Furthermore, males were more likely than females to seek treatment at private providers, which would have impacted the overall results with regards to treatment-seeking behaviour and costs [8]. Another group excluded from the survey were temporary migrants who had moved into the area within last six months and did not plan to stay longer than six months. Depending on where they moved from, these individuals might be more susceptible to malaria than the general population. The survey was also undertaken in the 4 most accessible and populous sub-districts whose population may not be representative of the those in the more remote sub-districts where access to health care is more difficult; therefore, results may slightly overestimate the proportion seeking treatment outside the home.

A common issue with all surveys aimed at describing treatment-seeking behaviour and case management for malaria, is the unknown malaria status of the individuals who reported having a fever in the past month. Including all fevers in the previous month, enables the potential capture of entire fever episodes; however, they may also result in significant recall bias due to the length of time between the episode and the survey. In order to triangulate these results, healthcare facility exit surveys were undertaken in which patients were interviewed immediately after their visit, thus minimizing the potential for bias and enabling an examination of costs specific to the type of malaria diagnosed. Any treatment or indirect costs occurring other than this healthcare visit, however, would not be captured by this survey and thus potentially impact its cost estimates.

This study was undertaken at a time of very high treatment failure to the recommended first-line anti-malarials [11]. The poor efficacy of available treatments may partly explain the high rates of reported fever in the preceding month and why a third of respondents did not access treatment outside of the home.

## Conclusions

In summary these findings highlight a high level of reported febrile illness, which is most apparent in young children and those from poor households. Despite the provision of free treatment in public health facilities, treatment-seeking in the private sector was higher than expected, with considerably higher costs. The household costs of *P. vivax* were similar to *P. falciparum*, and in view of the relapsing and recurrent nature of malaria in this location, the financial burden of both infections is likely to be considerable. Anti-malarial programmes implementing policy change of first-line treatment will need to engage both public and private sectors if early diagnosis and highly effective treatment is to impact on the whole community.

## Additional files

**Additional file 1: Fig. S1.** Results from the Discriminant Analysis of Principal Components to construct socio-economic status groupings. Description: **a** Shows the household income divided by the number of household members for each group (US$). **b** Shows the monthly household expenditure divided by the household members (US$) for each group. **c** Tree plot of ownership by groups from poorest (black) to richest (lightest grey).

**Additional file 2: Fig. S2.** Mean household expenditure on health by SES groups.

**Additional file 3: Table S1.** Median costs (US$) and interquartile range (IQR) by healthcare provider (corresponding to costs in Table 4).

**Additional file 4: Table S2.** Reported direct, indirect and total costs (including illness days) per individual taking treatment for fever over the entire fever episode (N=834).

**Additional file 5: Table S3.** Median and interquartile range (IQR) of patient costs in US$ per visit to a public provider by malaria diagnosis from facility exit surveys (corresponding to the costs in Table 6).

## Abbreviations

CI: confidence interval
IDR: Indonesian rupiah
IQR: interquartile range
OR: odds ratio
SES: socio-economic status
SD: standard deviation
SP: sulfadoxine-pyrimethamine
US$: United States dollar
