# Supplementary figures and images for: Treatment-seeking behaviour and associated costs for malaria in Papua, Indonesia
Source: Malar J. 2016 Nov 8;15:536. doi: 10.1186/s12936-016-1588-8 (PMC5100266; doi:10.1186/s12936-016-1588-8)

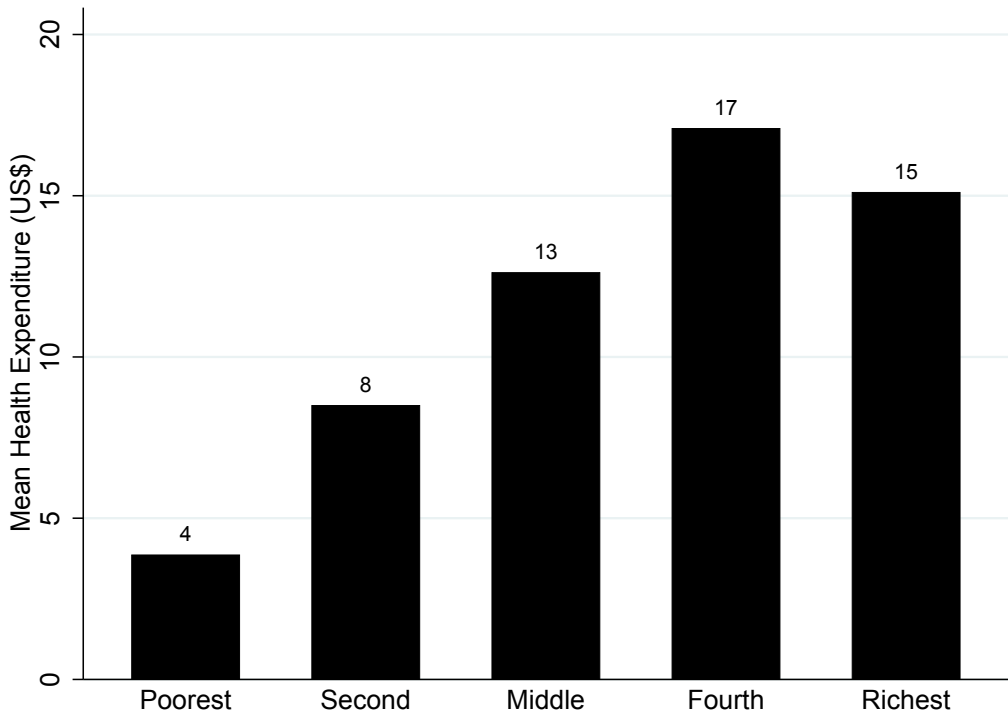

Supplement: Supplementary file 2 — Additional file 2: Fig. S2. Mean household expenditure on health by SES groups. [file 12936_2016_1588_MOESM2_ESM.pdf]
