# Supplementary material for: Treatment-seeking behaviour and associated costs for malaria in Papua, Indonesia
Source: Malar J. 2016 Nov 8;15:536. doi: 10.1186/s12936-016-1588-8 (PMC5100266; doi:10.1186/s12936-016-1588-8)
Supplement: Supplementary file 3 — Additional file 3: Table S1. Median costs (US$) and interquartile range (IQR) by healthcare provider (corresponding to costs in Table 4). [file 12936_2016_1588_MOESM3_ESM.docx]

**Table S1. Median costs (US$) and interquartile range (IQR) by healthcare provider (corresponding to costs in Table 4).**

| Healthcare provider | Visit cost in US$^a^ | | Transport cost in US$^b^ | | Total direct cost in US$ | |
| --- | --- | --- | --- | --- | --- | --- |
|  | Median | IQR | Median | IQR | Median | IQR |
| **Private (N = 519)** | 2.80 | 0.93 – 8.40 | 0.56 | 0.00 – 0.93 | 3.74 | 1.49 – 9.99 |
| Private clinic or doctor (N = 240) | 7.47 | 2.80 – 18.68 | 0.84 | 0.37 – 1.12 | 9.34 | 3.74 – 22.69 |
| Pharmacy (N = 173) | 1.87 | 0.93 – 3.92 | 0.56 | 0.37 – 0.75 | 2.61 | 1.68 – 5.04 |
| Shop (N = 106) | 0.75 | 0.37 - 1.49 | 0.00 | 0.00 – 0.19 | 0.84 | 0.37 – 1.87 |
| **Public (N = 375)** | 0.37 | 0.00 – 0.75 | 0.37 | 0.00 – 0.93 | 0.93 | 0.00 – 1.87 |
| Puskesmas (N = 155) † | 0.75 | 0.37 - 1.42 | 0.37 | 0.00 – 0.75 | 1.21 | 0.75 – 2.36 |
| Malaria control clinic (N = 149) †† | 0.00 | 0.00 – 0.00 | 0.00 | 0.00 – 0.56 | 0.00 | 0.00 – 0.56 |
| Hospital (N = 71) | 0.37 | 0.37 – 0.93 | 1.40 | 0.93 – 2.05 | 1.87 | 1.31 – 2.80 |

^a^ Includes costs of consultation, diagnosis, medications and any other costs directly related to the care received.

^b^ Includes costs for the patient and anyone who accompanied him or her.

† Government-funded primary health clinic

†† Mining company-funded clinic
