# Supplementary material for: Treatment-seeking behaviour and associated costs for malaria in Papua, Indonesia
Source: Malar J. 2016 Nov 8;15:536. doi: 10.1186/s12936-016-1588-8 (PMC5100266; doi:10.1186/s12936-016-1588-8)
Supplement: Supplementary file 4 — Additional file 4: Table S2. Reported direct, indirect and total costs (including illness days) per individual taking treatment for fever over the entire fever episode (N=834). [file 12936_2016_1588_MOESM4_ESM.docx]

Table S2. Reported direct, indirect and total costs (including illness days) per individual taking treatment for fever over the entire fever episode (N=834)

|  | Age | | | | | | | | Overall | | | |
| --- | --- | --- | --- | --- | --- | --- | --- | --- | --- | --- | --- | --- |
|  | Child (n=353) | | | | Adult (n=481) | | | |  |  |  |  |
| Category | Mean (SD) | Median | IQR | Mean (SD) | | Median | IQR | Mean (SD) | | Median | IQR |  |
| Total direct costs (including payments for all treatments) | 7.34 (25.72) | 1.31 | 0.19 – 5.04 | 10.83 (28.38) | | 1.96 | 0.41 – 6.16 | 9.35 (27.33) | | 1.87 | 0.37 – 5.60 |  |
| Total indirect costs | 31.49 (35.92) | 27.31 | 10.92 – 38.23 | 53.52 (58.06) | | 38.23 | 16.39 – 76.46 | 44.19 (51.05) | | 32.77 | 10.92 – 60.08 |  |
| Individual taking treatment |  |  |  |  | |  |  |  | |  |  |  |
| Number of days unable to perform all usual activities ^a^ | - | - | - | 1.59 (2.52) | | 0.00 | 0.00 – 3.00 | - | | - | - |  |
| Number of days unable to perform some usual activities ^a,b^ | - | - | - | 2.96 (4.23) | | 2.00 | 0.00 – 5.00 | - | | - | - |  |
| Total cost to the individual due to lost wages (US$) | - | - | - | 33.47 (33.95) | | 27.31 | 10.92 – 43.69 | 19.31 (30.63) | | 0.00 | 0.00 – 32.77 |  |
| All companions |  |  |  |  | |  |  |  | |  |  |  |
| Number of days unable to perform all usual activities | 0.17 (0.57) | 0.00 | 0.00 – 0.00 | 0.10 (0.38) | | 0.00 | 0.00 – 0.00 | 0.13 (0.47) | | 0.00 | 0.00 - 0.00 |  |
| Number of days unable to perform some usual activities ^b^ | 0.73 (0.77) | 1.00 | 0.00 – 1.00 | 0.30 (0.61) | | 0.00 | 0.00 – 0.00 | 0.48 (0.72) | | 0.00 | 0.00 – 1.00 |  |
| Total cost for companions (US$) | 5.86 (7.58) | 5.46 | 0.00 – 5.46 | 2.78 (5.21) | | 0.00 | 0.00 – 5.46 | 4.09 (6.50) | | 0.00 | 0.00 – 5.46 |  |
| All caretakers |  |  |  |  | |  |  |  | |  |  |  |
| Number of days unable to perform all usual activities | 0.31 (1.73) | 0.00 | 0.00 – 0.00 | 0.23 (1.55) | | 0.00 | 0.00 – 0.00 | 0.27 (1.63) | | 0.00 | 0.00 – 0.00 |  |
| Number of days unable to perform some usual activities ^b^ | 4.03 (4.41) | 3.00 | 1.00 – 5.00 | 2.36 (4.50) | | 0.00 | 0.00 – 3.00 | 3.07 (4.54) | | 1.00 | 0.00 – 5.00 |  |
| Total cost for caretaking (US$) | 25.45 (31.77) | 21.85 | 5.46 – 32.77 | 15.42 (28.92) | | 0.00 | 0.00 – 21.85 | 19.67 (30.54) | | 10.92 | 0.00 – 27.31 |  |
| All substitute labourers |  |  |  |  | |  |  |  | |  |  |  |
| Number of days unable to perform all usual activities | 0.00 (0.00) | 0.00 | 0.00 – 0.00 | 0.06 (0.40) | | 0.00 | 0.00 – 0.00 | 0.03 (0.30) | | 0.00 | 0.00 – 0.00 |  |
| Number of days unable to perform some usual activities ^b^ | 0.03 (0.36) | 0.00 | 0.00 – 0.00 | 0.22 (1.33) | | 0.00 | 0.00 – 0.00 | 0.14 (1.04) | | 0.00 | 0.00 – 0.00 |  |
| Total cost for substitute labour (US$) | 0.17 (1.95) | 0.00 | 0.00 – 0.00 | 1.84 (8.39) | | 0.00 | 0.00 – 0.00 | 1.13 (6.54) | | 0.00 | 0.00 – 0.00 |  |
| Total costs | 38.82 (46.41) | 29.18 | 16.39 – 45.35 | 64.34 (74.43) | | 40.47 | 16.39 – 87.39 | 53.54 (65.28) | | 33.75 | 16.39 – 66.66 |  |

^a^ Not reported by children.

^b^ Days reported as cutting back on some activities were included in the cost calculations as half of the mean wage per day.
