# Supplementary material for: Treatment-seeking behaviour and associated costs for malaria in Papua, Indonesia
Source: Malar J. 2016 Nov 8;15:536. doi: 10.1186/s12936-016-1588-8 (PMC5100266; doi:10.1186/s12936-016-1588-8)
Supplement: Supplementary file 5 — Additional file 5: Table S3. Median and interquartile range (IQR) of patient costs in US$ per visit to a public provider by malaria diagnosis from facility exit surveys (corresponding to the costs in Table 6). [file 12936_2016_1588_MOESM5_ESM.docx]

**Table S3. Median and interquartile range (IQR) of patient costs** in US$ per visit to a public provider by malaria diagnosis from facility exit surveys (corresponding to the costs in Table 6).

|  | P. falciparum (N=196) | | P. vivax (N=136) | |
| --- | --- | --- | --- | --- |
|  | Median | IQR | Median | IQR |
| Total direct costs | 1.42 | 0.84 – 2.25 | 1.31 | 0.00 – 2.24 |
| Visit cost | 0.37 | 0.00 – 0.75 | 0.37 | 0.00 – 0.37 |
| Transport cost for patient | 0.16 | 0.00 – 0.56 | 0.00 | 0.00 – 0.28 |
| Transport cost for companions | 0.56 | 0.00 – 1.12 | 0.28 | 0.00 – 1.17 |
| Total indirect costs ^a^ | 27.31 | 10.92 – 60.08 | 27.31 | 10.92 – 54.62 |
| Lost wages for patient ^b^ | 0.00 | 0.00 – 27.31 | 0.00 | 0.00 – 5.46 |
| Lost wages for companions | 5.46 | 0.00 – 10.92 | 5.46 | 0.00 – 10.92 |
| Lost wages for caretakers | 10.92 | 0.00 – 30.04 | 10.92 | 0.00 – 38.23 |
| Lost wages for substitute labourers | 0.00 | 0.00 – 0.00 | 0.00 | 0.00 – 0.00 |
| Total costs | 29.51 | 11.86 – 65.59 | 28.34 | 10.92 – 60.73 |

^a^ The per day wage was taken from the household survey (US$10.93).

^b^ For children, lost wages are recorded as no cost.
