# Supplementary material for: Treatment-seeking behaviour and associated costs for malaria in Papua, Indonesia
Source: Malar J. 2016 Nov 8;15:536. doi: 10.1186/s12936-016-1588-8 (PMC5100266; doi:10.1186/s12936-016-1588-8)
Supplement: Supplementary file 1 — Additional file 1: Fig. S1. Results from the Discriminant Analysis of Principal Components to construct socio-economic status groupings. Description: a Shows the household income divided by the number of household members for each group (US$). b Shows the monthly household expenditure divided by the household members (US$) for each group. c Tree plot of ownership by groups from poorest (black) to richest (lightest grey). [file 12936_2016_1588_MOESM1_ESM.pdf]

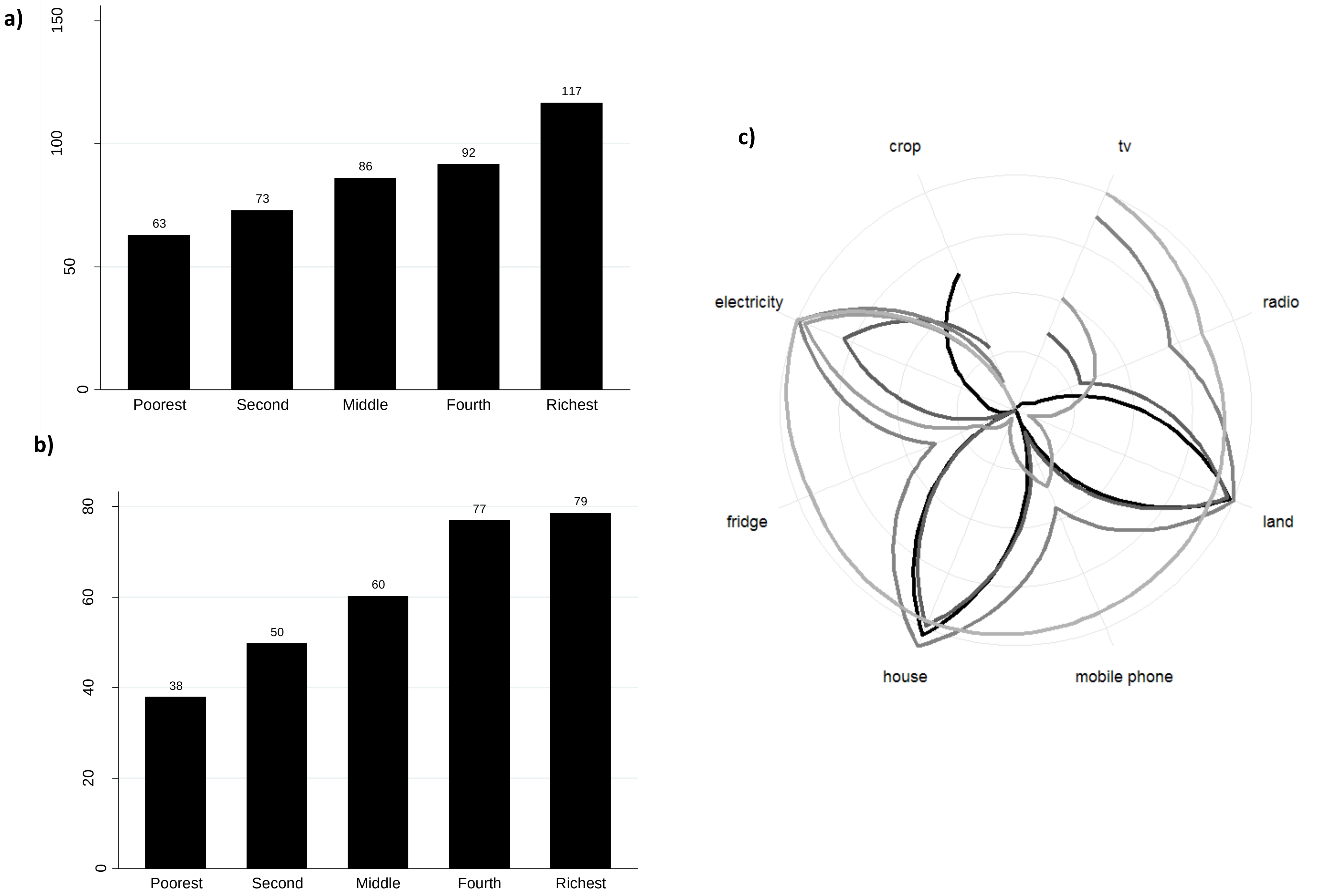
a)
150
100
50
0
63
73
86
92
117
Poorest
Second
Middle
Fourth
Richest
b)
80
60
40
20
0
38
50
60
77
79
Poorest
Second
Middle
Fourth
Richest
c)
crop
tv
electricity
radio
fridge
land
house
mobile phone
